# Case report: Fatal long-term intoxication by 2,4-dinitrophenol and anabolic steroids in a young bodybuilder with muscle dysmorphia

**DOI:** 10.3389/fpubh.2024.1452196

**Published:** 2024-11-26

**Authors:** Coralie Hermetet, Marine Jourdan, Alain Baert, Laurie Gheddar, Alice Ameline, Pascal Kintz, Renaud Bouvet

**Affiliations:** ^1^Department of Legal and Penitentiary Medicine, Rennes University Hospital, Rennes, France; ^2^Institute of Forensic Medicine, Strasbourg, France; ^3^Institute of Public Law and Political Science, University of Rennes, Rennes, France

**Keywords:** 2,4-dinitrophenol, muscle dysmorphia, bodybuilding, chronic poisoning, aggressive resuscitation therapeutics, case report

## Abstract

A case of chronic intoxication by 2,4-dinitrophenol (2,4-DNP) is reported in a 21-year-old bodybuilder, also known as an abuser of anabolic steroids, who died after ingesting 2 grams of this substance after 6 months of repeated consumption. The bodybuilder presented the triad of symptoms – tachycardia, tachypnoea, profuse sweating – from 6 months before his death, and was hospitalised for multiple organ failure 4 months before his death. Medical staff attributed this serious episode to his consumption of 2,4-DNP. Although the triad of symptoms persisted, he denied continuing to consume 2,4-DNP during consultations with his general practitioner, who therefore looked into a possible diagnosis of endocrine or tumour disorder. The bodybuilder died of multi-organ failure. The autopsy found diffuse visceral congestion and yellowish colouration of integuments. Toxicology demonstrated not only lethal acute 2,4-DNP intoxication (blood concentration was 88 mg/L), but also chronic intoxication (segmental hair concentrations were 5.1 to 25.5 ng/mg). Different anabolic steroids were also identified in the hair. Continued uncontrolled consumption of 2,4-DNP despite the consequences for his health, combined with an obvious preoccupation with his physical appearance, supported the suspected diagnosis of “muscle dysmorphia”, a psychiatric disorder in which dangerous substances are trivialised. Primary care professionals, the first to come into contact with intoxication cases, should receive training on how to detect and manage cases with symptomology that mimics 2,4-DNP use. A large study evaluating the outcomes of acute intoxication cases if “aggressive” management had been immediately implemented may help us determine the optimal course of action that minimises fatalities.

## Introduction

The pathophysiology of 2,4-dinitrophenol (2,4-DNP, chemical formula C_6_H_4_N_2_O_5_) has been explored since the 1930s (1): its lipophilic nature allows it to cross membranes to reach the mitochondrial matrix where it releases a hydrogen ion (H+), such that the H+ gradient is eventually abolished. Given that this gradient normally constitutes the proton-motive force for oxidative phosphorylation, the effect is to decouple oxidative phosphorylation and to interfere with the Krebs cycle. Furthermore, in the presence of 2,4-DNP, inorganic phosphate is no longer taken up by mitochondria, so it cannot be added to ADP molecules by ATP synthase. The energy released during oxidation–reduction reactions becomes insufficient to boost ATP production and is dissipated in the form of heat: as the dose of 2,4-DNP is increased, energy production gradually becomes less efficient, and metabolism increases to compensate for the lack of efficiency and meet energy requirements. The main clinical manifestations of 2,4-DNP intoxication are summarised in Table 1. The classic acute triad consists of: profuse sweating (non-systematic hyperthermia), tachycardia, and tachypnoea (2–4). In chronic intoxication, development of a cataract is the main manifestation (5). Another characteristic feature of exposure to 2,4-DNP is yellowish colouration of integuments due to impregnation by nitrate derivatives (6, 7). In cases of lethal intoxication, death occurs on average within 14 h of ingestion (4).

**Table 1:** Main clinical manifestations of acute and chronic intoxication by 2,4-dinitrophenol.

Acute intoxication (6)	Chronic intoxication (5, 6, 8)
General: hyperthermia, profuse sweating Cardiovascular: tachycardia, palpitations Gastrointestinal: nausea-vomiting, abdominal pain Neurological: headaches, confusion, agitation, dizziness Respiratory: dyspnoea, tachypnoea Renal: acute kidney disease (tubular necrosis)	Cutaneous: Fournier’s gangrene, maculopapular erythematous skin eruptions, rash Ophthalmological: cataract Haematological: bone marrow lesions, agranulocytosis Neurological: CNS lesions
Exposure: yellow colouration of integuments (6)	

Since 1916, scientific publications on acute and chronic 2,4-DNP intoxication have described the clinical course during the hospital stay, which sometimes culminated in death (3, 8, 9). However there do not seem to be any published articles presenting the clinical history of chronic consumers of 2,4-DNP. In addition, it is notably sportspeople who continue using 2,4-DNP despite its toxic effects (10), which prompts the question of whether a psychiatric comorbidity might be present in these consumers. The case study presented involves a 21-year-old male who had been consuming 2,4-DNP for several months but only reported this while being treated for serious adverse events in hospital 4 months before his death; he was receiving medical care throughout the period of exposure.

## Case presentation

### Medical history

**Figure 1:**
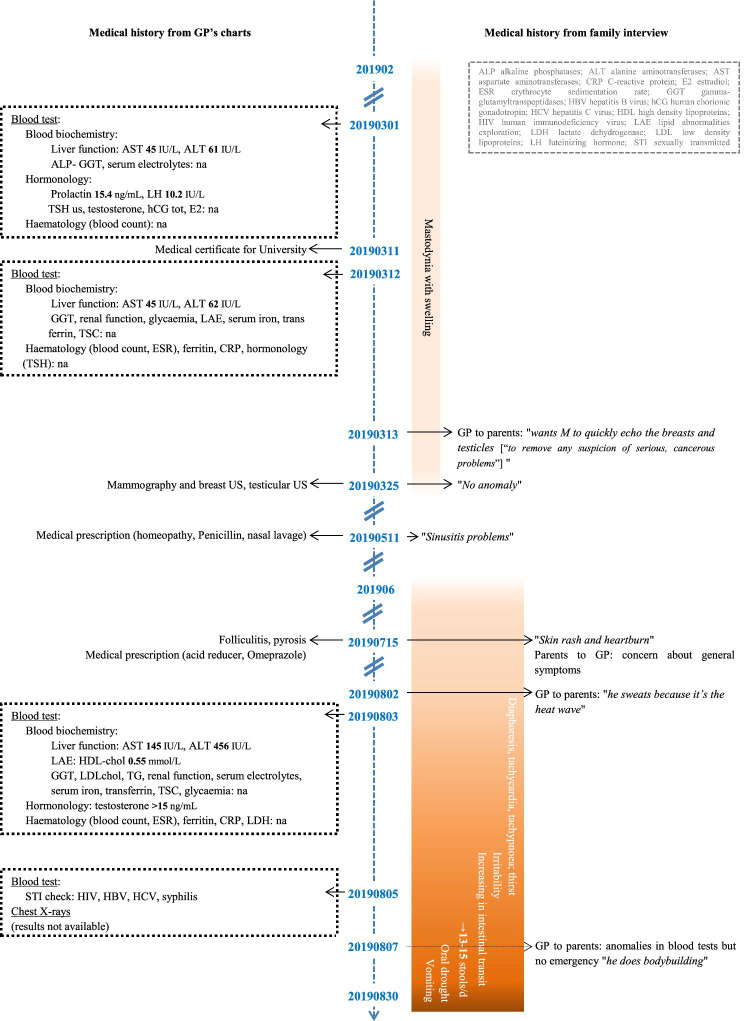
Timeline of clinical and laboratory/imaging findings for M, February to August 2019. GP, general practitioner; US, ultrasound; na, no abnormality.

Figure 1 shows clinical and laboratory/imaging findings available from February to August 2019. The young man (henceforth called M) had neither remarkable medical history since childhood nor weight gain issues, was up to date with all his vaccinations, had not traveled abroad in the year preceding his death, and no member of his family or circle of friends or fellow students had symptoms of a contagious infection. Since 2017, he had been under the care of the same primary care doctor (his GP), whom he first consulted in February 2019 with his first somatic complaint: painful gynaecomastia. Ultrasound examinations of mammary tissue and testes were unremarkable; blood panels showed hyperprolactinaemia, elevated LH, and hepatic cytolysis predominantly GPT. The symptoms resolved spontaneously. Symptoms developed from June 2019; sweating, followed by tachycardia, tachypnoea and a sensation of thirst. His GP did not fully appreciate the significance of the symptoms and laboratory abnormalities. Gastrointestinal symptoms and irritability then developed. During this period, M visited his GP several times and underwent additional investigations including blood testing (testosterone undetectable), testing for sexually transmitted infections (negative results) and a chest X-ray.

On 30 August 2019 at 4 am, M presented at A&E: symptoms were at their peak, and also included mouth dryness and vomiting. He had tachycardia of 145 bpm, systolic hypertension and moderate hyperthermia. Laboratory testing showed incipient acute kidney failure in association with rhabdomyolysis, which added to the compensated respiratory alkalosis. He was managed in the resuscitation room, then admitted. It was only fifteen hours after his admission that he disclosed to the doctor that he had consumed 2,4-DNP, which he had obtained from someone at his gym. An evaluation carried out during his 3-day hospital stay revealed a *Campylobacter jejuni* infection (see Supplemental material S1 for detailed information on clinical findings, investigations and treatments).

**Figure 2:**
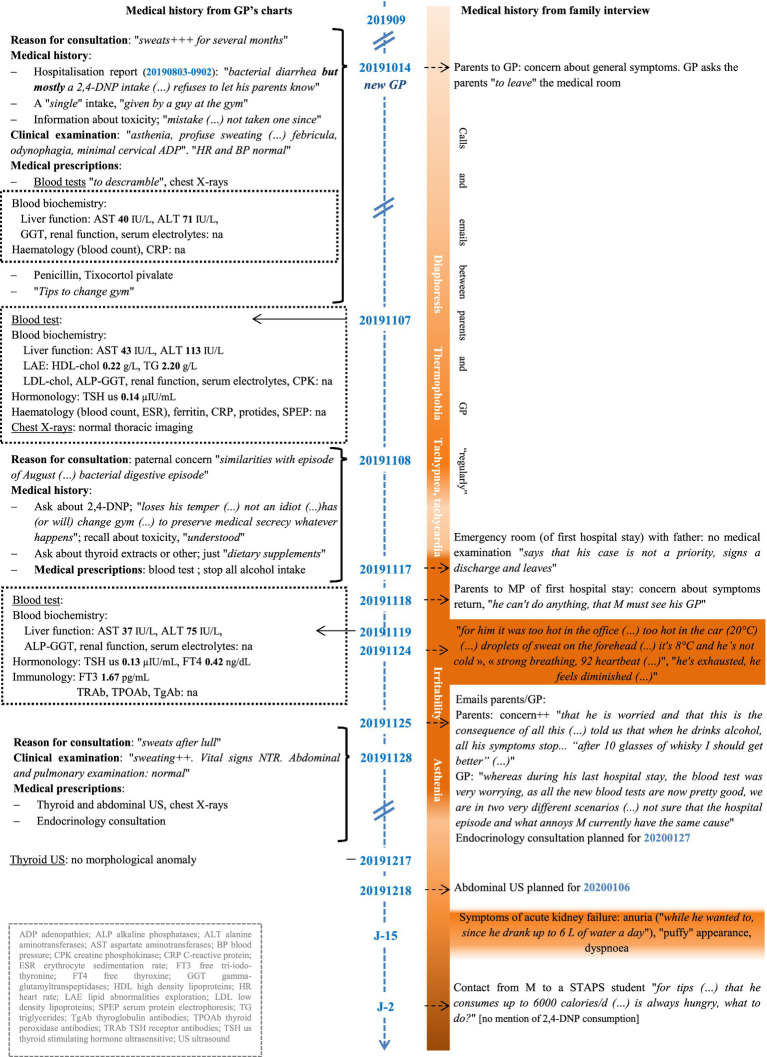
Timeline of clinical and laboratory/imaging findings for M, September 2019 to January 2020. GP, general practitioner; MP, medical practitioner; STAPS, sports science; US, ultrasound.

Figure 2 contains the clinical manifestations, investigations and treatments received from September 2019 to January 2020. In the first few weeks after being discharged from hospital, M developed many symptoms over time: profuse sweating, thermophobia, tachycardia, tachypnoea, then irritability, asthenia and symptoms suggestive of renal damage (oliguria, “puffed up” appearance). After returning home, he consulted the GP who had been treating him from childhood to adulthood. Between September and December 2019, as symptoms became worse, he saw his GP three times; several additional investigations were ordered and an appointment was scheduled with an endocrinologist for January 2020. Given the persistence and deterioration of his clinical presentation, on several occasions his GP raised the problem of 2,4-DNP consumption given that M had disclosed this during the August 2019 hospital stay. Although the discharge summary seemed to have established chronic consumption, M continued to admit a single use of 2,4-DNP. Thus, in consultations with his GP, he denied being a regular consumer of 2,4-DNP in the period before his August 2019 hospital stay and in the period following discharge.

On 4 January 2020, shortly after 2 am, M called the medical emergency number reporting a clinical picture similar to that of August 2019 and that he had consumed 2 g of 2,4-DNP approximately 4 h earlier. He arrived at the A&E of the university hospital approximately 6 h after ingestion (H + 6), at which time other than intense tachypnoea and respiratory alkalosis, there were no clear signs of organ failure; neither headaches, nor photo/phonophobia, nor hyperthermia. He was placed under surveillance. At around H + 9, the clinical picture deteriorated precipitously: worsening neurological symptoms (confusion, agitation) and tachycardia, systolic hypertension, temperature rising from 37°C to 38 then to 41°C despite the application of external cooling measures, with a fall in respiratory rate, multi-organ failure, development of ventricular tachycardia then asystole. At H + 11, axial hypertonia developed whose intensity was so severe as to hinder intubation and prevent mechanical ventilation. In view of the inefficacy of treatments initiated, the non-existence of an antidote and the persistent interruptions to the circulation, resuscitation efforts were terminated at H + 12. Axial hypertonia persisted postmortem (see Supplemental material S2 for detailed information on clinical findings, investigations and treatments).

### Postmortem investigation

The subject was a male Caucasian 183 cm tall and weighing 82 kg (BMI 24.5 kg/m^2^). At autopsy, the only notable findings were yellow colouration of the plantar surface of the feet and diffuse visceral congestion. Upon extraction and dissection, the brain was examined and found to be normal. The pathological examination of the heart (weight: 360 g, for a norm according to his morphology: 378 g [293; 463] (11)) revealed left ventricular and septal hypertrophy without architectural disorganisation. Microscopic examination of hepatic and renal samples was unremarkable. The toxicology analysis revealed the presence of 2,4-DNP in the blood (88 mg/L) and urine (83 mg/L) (12). Results of the toxicological hair analysis (total length of sample: 9 cm) are presented in Table 2. Tests for the presence and quantification of 2,4-DNP in hair by LC–MS/MS were recently published by Kintz and Ameline (13). Anabolic steroids were tested in hair using a previously published method and demonstrated repetitive abuse of various substances. With respect to the measured concentrations, they can be interpreted as corresponding to high levels of consumption over a long period of time, i.e., at least 9 months (14).

**Table 2:** Toxicological hair analysis.

Substances (units)	Hair concentrations by segment (/3 cm)		
	0 to 3 cm	3 to 6 cm	6 to 9 cm
2,4-dinitrophenol (ng/mg) (13)	5.1	13.1	25.5
AAS:			
Testosterone (pg/mg)	12	14	16
Testosterone enanthate (pg/mg)	Not detected	25	49
Androstenedione (pg/mg)	9	8	11
Boldenone (pg/mg)	1	1,5	2
DHEA (pg/mg)	11	13	15
Nandrolone (pg/mg)	14	10	10
Stanozolol (pg/mg)	40	45	44
Trenbolone (pg/mg)	360	215	267
Trenbolone enanthate (pg/mg)	6	25	35
Methandienone (pg/mg)	15	105	385
Clenbuterol (pg/mg)	47	178	76

AAS, anabolic–androgenic steroids; DHEA, dehydroepiandrosterone.

The coronial inquiry painted a picture of a young man without difficulties in his school/university, family or personal life, who visited several gyms on a daily basis and followed a high-protein diet in order to build muscle. Investigations at his home point to polyconsumption of various licit and illicit substances: anabolic steroids (stanozolol, beta-hCG, nandrolone, testosterone, trenbolone), protein preparations, and 2,4-DNP contained in sachets labelled “2,4-dinitrophenol; 100 capsules; 250 mg each capsule.” Within the detection limits of the Rennes University Hospital toxicology laboratory’s equipment (high-performance liquid chromatograph (HPLC) coupled with a diode array detector (Alliance, Waters), exact mass spectrometry), none of the NPS included in the databases at the time of the analyses were found in either the blood or the urine. The inquiry’s conclusion was accidental death.

## Discussion

### 2,4-DNP: an appearance-and performance-enhancing drug

Considered unsuitable for human consumption by the USA’s Food and Drug Administration since 1938 due to its side effects and the lack of therapeutic margin or antidote in case of intoxication, 2,4-DNP initially used as a weight loss aid continues to be used in certain industrial and scientific fields (15–17), and most intoxications are accidental, occupational or suicide attempts (4, 18). Human consumption surged in the mid-2000s due to the emergence of websites targeted at bodybuilders that presented 2,4-DNP as an appearance and performance enhancing drug (APED) almost unrivalled as a “fat burner”, producing weight loss of up to 1.5 kg per week (2, 4, 19). The most commonly cited APEDs are anabolic–androgenic steroids (AASs, prevalence of use estimated at 1–5% worldwide, higher in the USA (10)), human growth hormone (human GH, either from an exogenous source or by stimulating endogenous production via GHB consumption), creatine, blood doping agents such as erythropoietin (EPO), amphetamines and stimulants, and beta-hydroxy-beta-methylbutyrate (HMB) (20–22). The main effects sought by consumers of these substances are: increase in muscle mass (AASs); decrease in fat mass (AASs, human GH); improvement in physical performance (endurance, strength, speed) (creatine, amphetamines/stimulants, EPO, HMB); and acceleration of metabolism and improved vigilance (amphetamines/stimulants) (10, 21). In relation to the goal of losing fat mass, the effects of the more “traditional” APEDs only become visible after several weeks to several months of consumption in most cases in combination with intense physical exercise. 2,4-DNP is appealing as it seems to give faster and “easier” results (23). Despite its side effects and its impact on wellbeing, it is still viewed by consumers as requiring less effort than AASs (23). Polyconsumption is common among users of APEDs. Chronic consumption of AASs by M was confirmed by hair analysis (see Table 2) and was probably linked to the pathological finding of left ventricular hypertrophy not attributable to hypertrophic cardiomyopathy, a frequently observed finding in cases of AAS impregnation (24). It was not possible to measure AASs in M’s blood taken during his treatment at the beginning of January 2020 as the limited quantity of blood in the samples was used for detection and quantification of possible drugs and narcotics including 2,4-DNP. Nevertheless, literature data indicate that in the case of AASs, hair analyses prove to be more efficient than blood or urine tests (14). His lack of faith in his GP seems to be consistent with studies carried out among AAS users, who mainly evoke their concern about a judgemental attitude or discrimination, lack of confidence in their GP’s knowledge and concern about the legal ramifications (10).

Regarding the potential for toxicity and fatalities, most APEDs cause severe side effects in the intermediate and long term after chronic consumption, rather than after occasional exposure (cardiovascular pathology with AASs, pseudotumor cerebri with human GH). Amphetamines cause serious adverse events in the short term (arrhythmia, convulsions, sudden death); incidentally, some of these events are similar to those of acute intoxication by 2,4-DNP (21, 24). 2,4-DNP appears to be one of the most dangerous APEDs in the short term, with lethal ingested doses reported in the literature ranging from “300 mg in 6 weeks” to a one-shot 5 g, and death being primarily due to hyperthermia (4, 25). A resurgence of intoxication cases, some with fatal outcome, has been observed since 2010, with signals essentially from the English-speaking world but also Northern and Eastern Europe and Italy. This prompted the World Health Organization to request information from poisons centres at the end of 2020 in order to understand the extent of 2,4-DNP poisoning by the systemic route. With the aid of this data it should be possible to implement action plans. The results of this survey were published in late 2021 (26, 27).

There are many websites selling 2,4-DNP and their owners have become proficient at shutting down websites and opening new ones as necessary. Though 2,4-DNP is available in pills or capsules, the text “not for human consumption” also appears systematically. Most of these websites are hosted on servers located outside the European Union, so this mention is sufficient to allow them to sell as much of the molecule as they want (2); and as soon as one website shuts down, others appear. Some studies have described 2,4-DNP consumers as informed and prepared for the side effects (2, 23); assertions like these seem to be unaware of the powerful denial in particular populations such as those with MD, and the trivialisation of these effects by consumers (4, 23); these factors complicate awareness-raising and prevention campaigns (28). Prevention campaigns warning about dangers would be effective among people who had never consumed 2,4-DNP, but not among regular consumers (29). Finally, primary care professionals themselves, including GPs, have little knowledge about this substance, and without a minimum level of curiosity, they may minimise side effects, even short-term ones (30). In view of the well-documented recent increase in fatal intoxication cases (26), it is necessary to strike a balance between prevention and communication involving targeted action by investigation teams, prevention messages targeted at people going to fitness centres and gyms, and better surveillance of online markets.

### Management of acute 2,4-DNP poisoning

There is no risk-free dose or duration of 2,4-DNP use. There is no antidote either. So, in the case of M as in previously published cases medical teams could not do anything to combat the major hyperthermia or hypertonia. Several approaches to managing hyperthermia have been explored, with little success (9, 31). M’s body temperature rose to 41°C, which appears slightly higher than the median temperature of subjects who died by 2,4-DNP intoxication (40.3°C) (32). Hypertonia also significantly contributed to death as it made mechanical ventilation impossible. Note that as the hair toxicological analyses demonstrated co-consumption of 2,4-DNP, AASs and clenbuterol, the contribution of these other substances to M’s death may not be formally excluded. Nevertheless the clinical presentation upon arrival in the emergency room and during his treatment including the “profuse sweating – tachycardia – tachypnoea” triad, followed by the onset of hyperthermia and hypertonia, attest to the major contribution of acute 2,4-DNP poisoning to multi-organ failure and ultimately death.

It seems that “aggressive” management from the outset, without waiting for decompensation, is not an approach frequently adopted by intensive care units. The usefulness of implementing an immediately aggressive approach as a matter of routine remains controversial, all the more so given that in most cases there is a lucid interval between the onset of the acute manifestations leading to hospital admission and the runaway metabolism leading to multi-organ failure (3, 8). Taking into consideration that nomograms are available for molecules similar to 2,4-DNP, for example 4,6-dinitro-o-cresol, and that the bioavailability of 2,4-DNP following ingestion is known, then it seems that it would be possible to use the patient’s reported dose to estimate a toxicity threshold to inform the decision on whether it is warranted to institute such aggressive management, which would include, for example, deep sedation with mechanical ventilation and IV injection of a curare-type muscle relaxant until the critical period had passed (3, 27, 33, 34).

### Muscle dysmorphia

After M’s death, his parents met his friends and student peers and learnt that, in the weeks before his death, he expressed concerns about his health, concerns that became ever more intrusive over time; side effects of 2,4-DNP intoxication were also behind multiple absences. He was also concerned about the inability of doctors to explain his health problems, seemingly never making the link between his symptoms and his chronic use of 2,4-DNP. His reactions to questioning by his GP point towards denial of this link. All these elements strongly suggest a diagnosis of muscle dysmorphia (MD), a diagnosis discussed by one of the authors of this report (MJ, MD specialised in forensic psychiatry) (35). MD, formerly called “reverse anorexia” (36), is described in DSM-5 as an obsessive-compulsive disorder in which the patient has a compulsive need to exercise or is even exercise-dependent. Among the three diagnostic criteria laid down by Olivardia in 2001 (37), M seemed to present at least two: criterion A: excessive preoccupation with the idea that one’s body is not sufficiently lean and muscular (this criterion is inferred from M’s attendance of “several gyms on a daily basis” combined with a “high-protein diet in order to build muscle”); and criterion B: consequences on one’s emotional and social life [M’s visits to gyms amounted to several hours per week and he continued taking the substances despite the consequences]. The fact that M denied that he was continuing to take 2,4-DNP could be the manifestation of a cognitive distortion whereby dysmorphophobia and fears of losing muscle mass are of greater concern than the deterioration of his health.

According to Pope et al., MD affects at least one in ten male bodybuilders (38). There are different tools for diagnosing MD, including those cited by Garcia-Rodriguez et al. (for example the Drive for Muscularity Scale). Three correlates of MD were found in AAS users: preoccupation with one’s body (criterion A of Olivardia), abandonment of leisure activities (criterion B) and avoidance of showing one’s body; in addition, four variables have been noted as relevant in predicting APED use: internalisation of a muscular ideal, preoccupation with one’s body (dissatisfaction), MD symptoms and doing sport (39). Young men who take up the most intensive muscle-building activities, such as bodybuilding, would presumably be those who were most dissatisfied with their musculature and so at higher risk of consuming APEDs (35). The desire to lose weight appears to be one of the factors that drives people who strive to attain the body image ideal to consume 2,4-DNP, even if they are aware of the risk of death (40). According to Cuadrado et al., “*psychopathology* [of MD was] *still unknown*” in 2018, and there were no programmes to prevent this specific dysmorphophobia; nevertheless several avenues, combining medical and social sciences, are under study (41).

## Conclusion

- 2,4-DNP is an APED widely available online with a documented rise in fatal intoxication cases since the early 2010s.
- Bodybuilders are particularly affected by APED consumption, and may also be affected by muscle dysmorphia that can lead to denial of medical issues that develop and the risks incurred by consuming APEDs.
- It seems necessary to educate primary care professionals who are most likely to interact with intoxication cases (GPs, sports doctors, emergency doctors), so that they are better able to detect and manage acute or chronic intoxication.

## Data Availability

The original contributions presented in the study are included in the article/Supplementary material, further inquiries can be directed to the corresponding author.
