# Supplementary material for: Case report: Fatal long-term intoxication by 2,4-dinitrophenol and anabolic steroids in a young bodybuilder with muscle dysmorphia
Source: Front Public Health. 2024 Nov 26;12:1452196. doi: 10.3389/fpubh.2024.1452196 (PMC11628266; doi:10.3389/fpubh.2024.1452196)
Supplement: Supplementary file 1 [file Image_1.pdf]

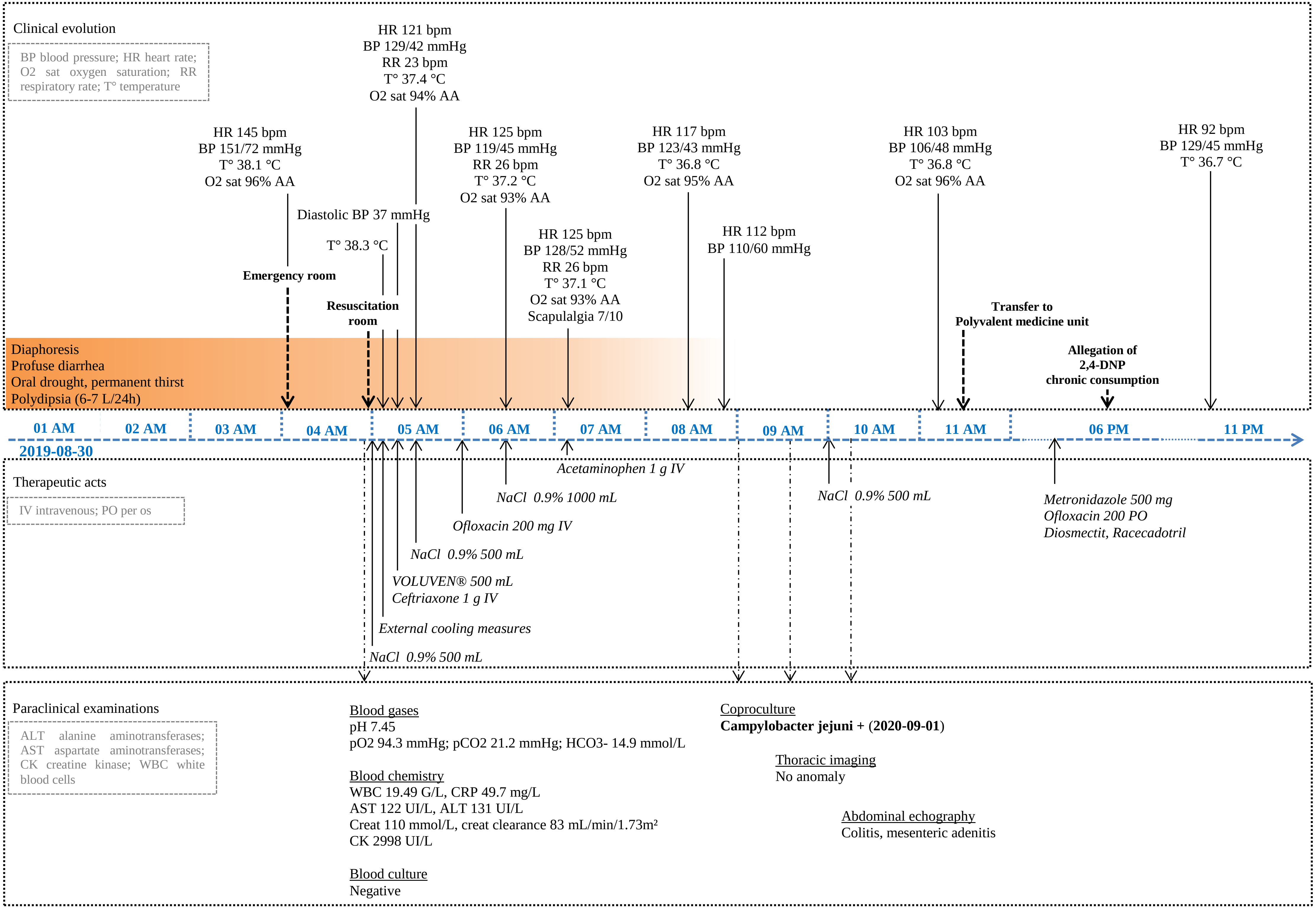

Clinical evolution
BP blood pressure; HR heart rate; O2 sat oxygen saturation; RR respiratory rate; T° temperature
HR 121 bpm
BP 129/42 mmHg
RR 23 bpm
T° 37.4 °C
O2 sat 94% AA
HR 145 bpm
BP 151/72 mmHg
T° 38.1 °C
O2 sat 96% AA
HR 125 bpm
BP 119/45 mmHg
RR 26 bpm
T° 37.2 °C
O2 sat 93% AA
HR 117 bpm
BP 123/43 mmHg
T° 36.8 °C
O2 sat 95% AA
HR 103 bpm
BP 106/48 mmHg
T° 36.8 °C
O2 sat 96% AA
HR 92 bpm
BP 129/45 mmHg
T° 36.7 °C
Diastolic BP 37 mmHg
T° 38.3 °C
HR 125 bpm
BP 128/52 mmHg
RR 26 bpm
T° 37.1 °C
O2 sat 93% AA
Scapulalgia 7/10
HR 112 bpm
BP 110/60 mmHg
Emergency room
Resuscitation room
Transfer to Polyvalent medicine unit
Allegation of 2,4-DNP chronic consumption
Diaphoresis
Profuse diarrhea
Oral drought, permanent thirst
Polydipsia (6-7 L/24h)
01 AM
02 AM
03 AM
04 AM
05 AM
06 AM
07 AM
08 AM
09 AM
10 AM
11 AM
06 PM
11 PM
2019-08-30
Therapeutic acts
IV intravenous; PO per os
Acetaminophen 1 g IV
NaCl 0.9% 1000 mL
Ofloxacin 200 mg IV
NaCl 0.9% 500 mL
VOLUVEN® 500 mL
Ceftriaxone 1 g IV
External cooling measures
NaCl 0.9% 500 mL
NaCl 0.9% 500 mL
Metronidazole 500 mg
Ofloxacin 200 PO
Diosmectit, Racecadotril
Paraclinical examinations
ALT alanine aminotransferases; AST aspartate aminotransferases; CK creatine kinase; WBC white blood cells
Blood gases
pH 7.45
pO2 94.3 mmHg; pCO2 21.2 mmHg; HCO3- 14.9 mmol/L
Blood chemistry
WBC 19.49 G/L, CRP 49.7 mg/L
AST 122 UI/L, ALT 131 UI/L
Creat 110 mmol/L, creat clearance 83 mL/min/1.73m²
CK 2998 UI/L
Blood culture
Negative
Coproculture
Campylobacter jejuni + (2020-09-01)
Thoracic imaging
No anomaly
Abdominal echography
Colitis, mesenteric adenitis
