# Supplementary figures and images for: Case report: Fatal long-term intoxication by 2,4-dinitrophenol and anabolic steroids in a young bodybuilder with muscle dysmorphia
Source: Front Public Health. 2024 Nov 26;12:1452196. doi: 10.3389/fpubh.2024.1452196 (PMC11628266; doi:10.3389/fpubh.2024.1452196)

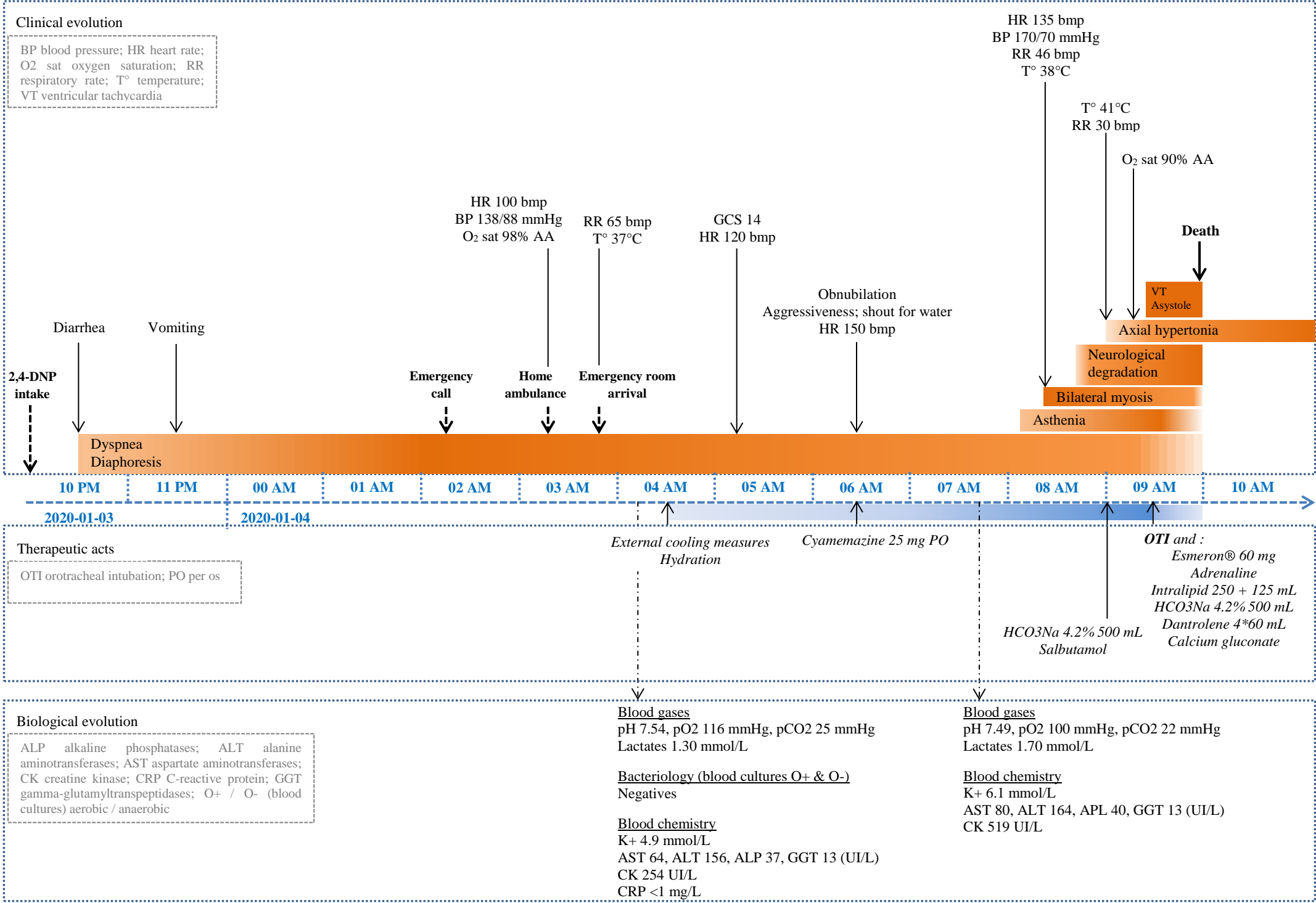

Supplement: Supplementary file 2 [file Image_2.pdf]
